# Anatomic Guidance For Ablation: Atrial Flutter, Fibrillation, and Outflow Tract Ventricular Tachycardia

**Published:** 2010-08-10

**Authors:** Nandini Sehar, Jennifer Mears, Susan Bisco, Sandeep Patel, Nirusha Lachman, Samuel J Asirvatham

**Affiliations:** 1 Division of Cardiovascular Diseases, Mayo Clinic, Rochester, Minnesota; 2 Department of Internal Medicine, Mayo Clinic, Rochester, Minnesota; 3 Department of Anatomy, Mayo Clinic, Rochester, Minnesota; 4 Department of Pediatrics and Adolescent Medicine, Mayo Clinic, Rochester, Minnesota

**Keywords:** atrial flutter, atrial fibrillation, anatomy, ablation, electrophysiology

## Abstract

After initial documentation of excellent efficacy with radiofrequency ablation, this procedure is being performed increasingly in more complex situations and for more difficult arrhythmia. In these circumstances, an accurate knowledge of the anatomic basis for the ablation procedure will help maintain this efficacy and improve safety.  In this review, we discuss the relevant anatomy for electrophysiology interventions for typical right atrial flutter, atrial fibrillation, and outflow tract ventricular tachycardia.  In the pediatric population, maintaining safety is a greater challenge, and here again, knowing the neighboring and regional anatomy of the arrhythmogenic substrate for these arrhythmias may go a long way in preventing complications.

## Introduction

Perhaps in no other subspecialty field in cardiology is the requirement for appreciating the detailed regional cardiac anatomy more significant than in cardiac electrophysiology. There is hardly an arrhythmia where either ablation safety or efficacy or both is not enhanced when the underlying anatomy is better appreciated. When performing procedures for complex arrhythmia and in high risk patient populations, anatomic knowledge becomes mandatory. In this review, we describe the anatomic basis for presently performed ablation procedures for atrial flutter, atrial fibrillation, and outflow tract ventricular tachycardia. The impact of anatomy knowledge for avoiding complications, particularly in the pediatric population, is emphasized for each of these arrhythmias [1-5].

## Typical Atrial Flutter

Although the anatomy for typical atrial flutter is best understood and is described in detail below, there are several generalizable concepts learnt from appreciating the anatomic trickle relationship from this relationship to other types of atrial flutter (atypical atrial flutter) and also when typical atrial flutter occurs in atypical situations (congenital heart disease) [6-10].

### Regional Anatomy

**Figure 1:**
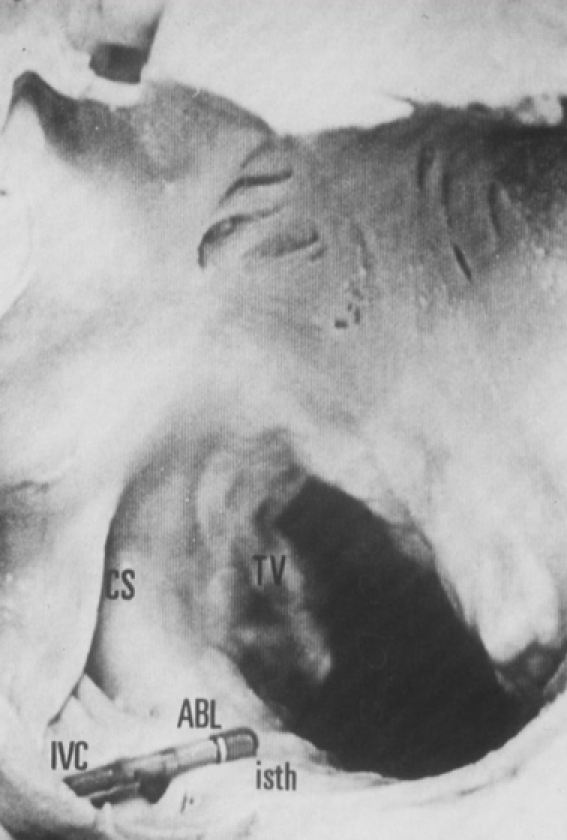
What the ablationist sees in his/her eyes when picturing the cavotricuspid isthmus.  Note the electrically inert tricuspid valve and what we expect to be a relatively flat terrain between the tricuspid valve and inferior vena cava.  TV - tricuspid valve; isth - isthmus; ABL - ablation; CS - coronary sinus; IVC - inferior vena cava.

**Figure 2:**
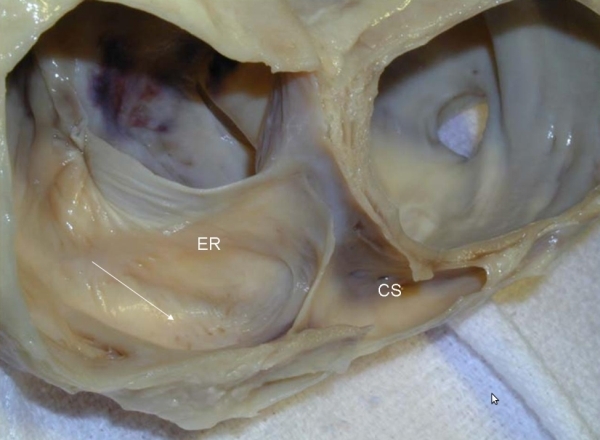
A more realistic view from an actual autopsied heart.  The complex regional anatomy of the cavotricuspid isthmus.  The arrow points to the sub-Eustachian pouch.  Note the prominent Thebesian valve guarding the opening of the coronary sinus (CS).  A prominent Eustachian ridge (ER) and valve associated with the Eustachian ridge are also visualized.

The atrial myocardium between the electrically inert tricuspid valve and inferior vena cava (IVC) (the cavotricuspid isthmus [CVTI]) is the anatomic target when ablating typical atrial flutter.  The CVTI varies in terms of length, width, and myocardial thickness from patient to patient and also within the same patient between the septal and free wall locations [11-15].  Ablation of the CVTI is often straightforward with linear or spot lesions being given in a continuous fashion between the tricuspid valve and IVC (Figure 1).  However, anatomic complexity of the CVTI in some patients creates considerable difficulty with complete and permanent ablation. The Eustachian ridge (ER), sub-Eustachian pouches, prominent pectinate muscles, and combinations of these anatomic variants, particularly yield complexity (Figure 2).

#### Sub-Eustachian Ridge

The ER along with the Eustachian valve helps in fetal life to direct oxygenated blood from the IVC through the foramen ovale to the left atrium. The ER transects the CVTI into an anterior sub-Eustachian and a posterior post-Eustachian isthmus. The sub-Eustachian isthmus generally consists of circumferentially arranged atrial myocardial fibers between the base of the ridge and the tricuspid valve. The ridge itself varies in terms of prominence and can be very well developed in some adult hearts. The ratio of myocardium to fibrous tissue in the ridge is also variable and in part, determines the conduction properties of this structure.  It should be emphasized, however, that in the majority of hearts, myocardium is present in the ER, and thus, ablation purely between the tricuspid valve and the ER cannot be expected to be universally efficacious [7,16,17].

The ER may be a source of difficulty when ablating the CVTI for several reasons:

- A prominent ER produces counterintuitive movements when manipulating the ablation catheter from the femoral route. Usually, clockwise torque applied on the catheter will result in septal orientation of the ablating electrode. However, when a prominent ER is present, the ridge acts as a fulcrum, and clockwise torque will result in the ablation electrode pointing away from the septum. This can make it difficult and necessitate the use of a guiding sheath that is placed distal to the ER.
- Although conduction is frequently present across the ridge, there can be in some cases a block across the ridge along portions of the CVTI. Thus, longitudinal dissociation of conduction along portions of the CVTI may result. The electrophysiologist needs to be aware of this possibility since bidirectional conduction block may be misdiagnosed with multielectrode catheters placed (one portion of these must be anterior to the ridge) whereas conduction may occur across the ridge, albeit slowly to the flutter circuit behind the ridge. Less commonly and almost always only seen when prior ablation has extensive prior ablation is performed, longitudinal dissociation of conduction within the sub-Eustachian isthmus may also be seen.  This likely has resulted from linear ablation lesions being created parallel to the annulus rather than across the CVTI.
- The ER is usually less prominent more laterally, and when a particularly prominent ridge is present, lateral CVTI ablation may be beneficial.

### Sub-Eustachian Pouches

In all patients, the sub-Eustachian portion of the CVTI is relatively inferior to the ER. However, in some patients, there is an actual 'excavation' of myocardium producing an aneurysmal-type dilation in the sub-Eustachian region called a pouch [7,14,18-21]. When pouches occur, they tend to be closer to the coronary sinus ostium than the free wall of the right atrium and can vary significantly in the depth as well as the anteroposterior dimension [7,11,22]. There also appears to be an association, possibly developmental, between prominence of Eustachian pouches and a prominent Thebesian valve guarding the opening of the coronary sinus. Thus, it is unusual to find a large sub-Eustachian pouch without significant evidence of a Thebesian valve.

There are several implications that arise as a result of a sub-Eustachian pouch when ablating the CVTI:

- Catheter contact may not occur within the depths of the pouch resulting in incomplete transaction of the CVTI.
- When catheter contact does occur, power delivery is often limited by impedance rise and coagulation formation as a result of poor blood flow within the pouch. Thus, larger electrode surface area or irrigation may be required when ablating in the depths of the pouch. However, care to avoid injury to the right coronary artery or perforation is required.
- To avoid possible complications, the ablation line can be done more laterally (8 o'clock to 9 o'clock on the LAO view), making use of the anatomic fact that the pouches are deepest and most likely to occur septally close to the coronary sinus ostium.
- In rare cases, circumferential 'isolation' of the pouch and then connecting the circular lesion both to the tricuspid valve and posteriorly to the IVC can be attempted.

### Prominent Pectinate Muscles

**Figure 3:**
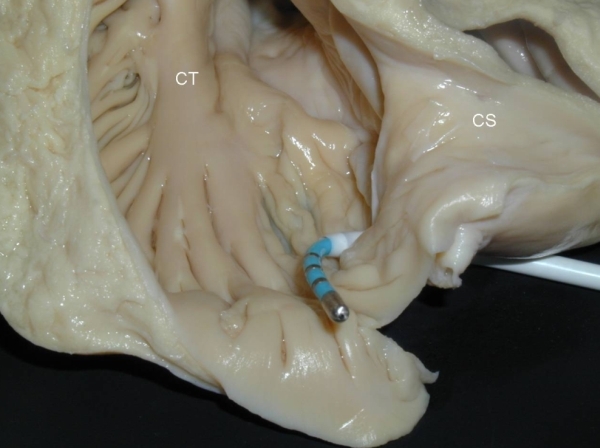
Dissection of an autopsied heart showing left anterior oblique-like view looking through the tricuspid valve into the right atrium with an ablation catheter and guiding sheath placed across the CVTI.  Note the large pectinate muscles emanating from the crista terminalis (CT).  Many pectinates are seen encroaching onto the CVTI and traversing the isthmus into the coronary sinus (CS).

In classical anatomy studies, the pectinate muscles are thought to emanate or 'terminate' on the crista terminalis in the posterolateral right atrium. The pectinates, however, frequently fan out from the crista onto the sub-Eustachian isthmus and at times, reaching within the coronary sinus (Figure 3).  In addition, pectinates may not terminate in the crista, but rather cross the structure and then end in a secondary, more medial and posterior ridge [22].

When present on the CVTI, the pectinates are most prominent laterally and gradually decrease in thickness as they traverse across the CVTI towards the ostium of the coronary sinus [7,23].

The anatomy of these pectinate extensions has several implications for the interventional electrophysiologist:

- When ablating on a pectinate muscle, energy delivery may not be sufficient to create a transmural lesion leaving a gap at this site. The situation can be compounded because of superficial fragmented electrograms found at a redo procedure, suggesting that further ablation may not be needed at that site. High amplitude electrograms prior to ablation delivery should suggest the presence of a pectinate muscle and energy delivery maximized at that site or an alternative position for the line considered.
- When the ablation electrode falls between two pectinate muscles, there can be an abrupt decrease in power and increase in impedance with or without coagulation formation since the electrode may be trapped in the crevice between the pectinates, limiting the effects of blood cooling the electrode. Larger electrode surface area (8 or 10-mm tip) or irrigation (open or closed) can help ablating over and between the pectinates [24].
- Since the pectinates are most prominent laterally, when their presence is recognized (large amplitude electrograms or ultrasound imaging), the physician can place the ablation line more septally close to the coronary sinus ostium. Care, however, should be taken when doing a very septal line that the catheter tip is not inadvertently within the middle cardiac vein since damage to the adjacent arterial vasculature may occur.
- When a posterior ridge septal to the crista terminalis is present, double potentials may be found in that location which the electrophysiologist should not misinterpret as a region of scar [25]. Such a ridge may also form a posterior boundary for the flutter circuit.
- When pectinates or other muscle bands are traversing posterior to the IVC beyond a posterior ridge, they may serve as a conduit for electrical conduction that may be utilized in a flutter circuit around the IVC (lower loop reentry). More commonly, however, these muscle bundles may allow passive activation of the free wall of the right atrium, giving the appearance of continued medial to lateral conduction despite adequate ablation of the CVTI.  Less commonly, these bundles serve as a conduit for activation going around the posterior IVC and then anteriorly onto the CVTI from a lateral to septal direction that gives the appearance of medial to lateral block across the isthmus even when slow conduction occurs (pseudo block). Careful high density maps done along the CVTI and posterior to the IVC will help clarify the situation [18,26,27].

### Atypical Flutter and Atypical Situations Where CVTI-Dependent Flutter Occurs

The ability to correlate anatomic lessons that underlie difficult CVTI ablation can be useful in atypical situations.

CVTI-dependent flutter is one of the most common macroreentrant tachycardias that occur in patients with congenital heart disease following surgery. The isthmus, however, may be transected by placement of a patch or conduit, and in some instances, the isthmus may be between the mitral annulus and the IVC (for example, in patients with cc-TGA - congenitally corrected transposition of the great vessels) [6,28-30].

When approaching an atypical flutter, electrophysiologists generally use a combination of entrainment and activation mapping to identify the critical or slow zone of the circuit and ablate at that site. An important lesson from the anatomy of the CVTI-dependent flutter circuit is that a slow zone as such may not necessarily be found. However, when the circuit can be proven to traverse through any two electrically inert structures, ablation can be done across that structure and bidirectional block used as an endpoint for the ablation line.

## Atrial Fibrillation

Although atrial fibrillation ablation in the pediatric population is uncommon, the principles of safe ablation in the left atrium including when isolating the pulmonary veins is dependent on appreciating the anatomic idiosyncrasies of the left atrium, pulmonary veins, and the adjacent noncardiac strucures [4,5,31].

Electrophysiologists should thoroughly understand both the necessity as well as the difficulties associated with knowing exactly where the pulmonary vein ostium is located.  In addition, one needs to be continuously cognizant of where the coronary vasculature and the esophagus are when delivering radiofrequency energy in the left atrium.

### Pulmonary Vein Ostium

**Figure 4:**
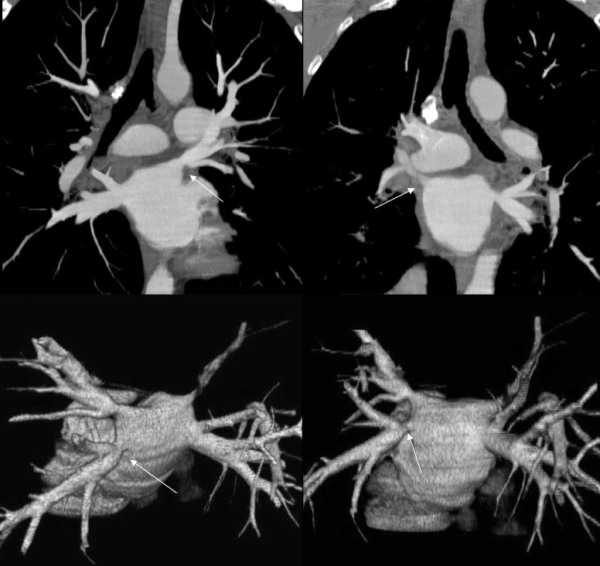
Computerized-tomographic images illustrating pulmonary vein stenosis.  Note the abrupt stenosis just into the pulmonary vein.  The lower images represent three-dimensional reconstructions. Pulmonary vein stenosis will not result if ablation delivery is kept proximal to the pulmonary vein ostium.  Arrow points to pulmonary vein stenotic areas.

An important and largely avoidable complication of AF ablation is pulmonary vein stenosis. A key anatomic concept to be borne in mind is that if ablation energy is not delivered within the pulmonary vein, then pulmonary vein stenosis will not occur (Figure 4). Although this concept is simple, it is an exceedingly difficult one to follow, especially when one is just becoming familiar with the AF ablation technique [26,32-34].

Anatomically, there is no specific structure that defines the pulmonary vein ostium (there are no valves or ridges, etc.). Thus, since even an anatomist cannot discern where the ostium of the pulmonary vein is, the proceduralist should always temper any information provided from advances in technology including merging of CT data or echocardiographic data to electroanatomic maps, etc. In other words, none of these modalities can tell the interventionalist where the ostium is, but on the contrary, it is the electrophysiologist who must tell the mapping system where the ostium is located.

**Figure 5:**
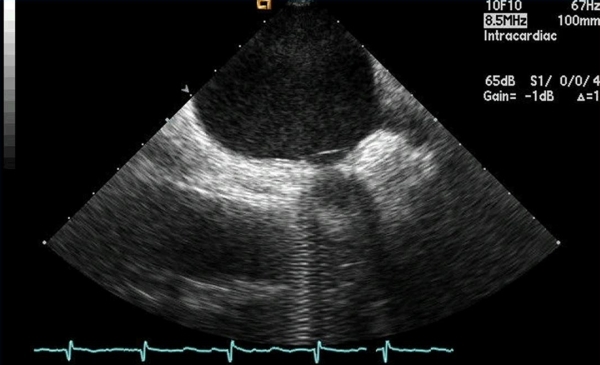
Intracardiac ultrasound image showing the circumferential mapping catheter (arrow) exactly at the ostium of the pulmonary vein.  Especially for the left-sided veins, ultrasound imaging can be helpful in approximating where the pulmonary vein ostium is located.  LA - left atrium

What information is helpful in trying to make this anatomic decision - where is the pulmonary vein ostium? As a generalization with CT merged data or intracardiac ultrasound, there often appears to be a transition between the cylindrical pulmonary veins and the more globoid left atrium (Figure 5). However, the presence of cloaca, common ostia for multiple veins, and funnel-shaped pulmonary veins make this an inexact method of defining the ostium [35].

Fluoroscopically, when a catheter is in the pulmonary vein, it may appear outside the cardiac silhouette. However, the exact fluoroscopic view being used largely determines the accuracy of such assumptions. For example, one can be quite deep in the right pulmonary veins in the left anterior oblique projection and yet appear within cardiac silhouette.

**Figure 6:**
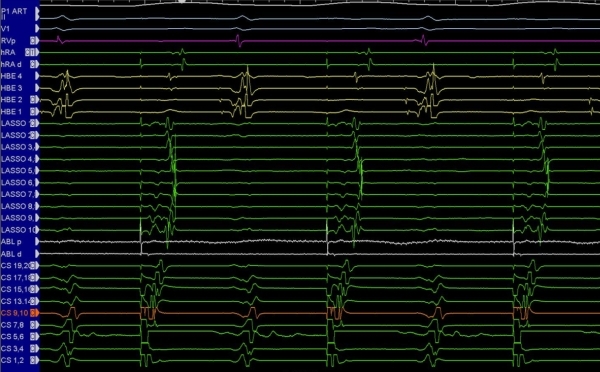
Intracardiac electrograms obtained from a left-sided pulmonary vein.  The lasso, a circumferential mapping catheter, has been placed into the vein.  Note the far-field atrial signals, isoelectric period, and the sharp near-field pulmonary vein potentials.  If an ablation catheter shows such a signal, then it is likely located inside the pulmonary vein.  Coronary sinus pacing is being performed.  CS - coronary sinus; HBE - His bundle recorded catheter; HRA - right atrial catheter; RV - right ventricular catheter; V1 and II - electrocardiographic leads.

The electrograms obtained when within the pulmonary vein versus when within the left atrium as well as the response to pacing maneuvers can be very helpful to the electrophysiologist when attempting to define the pulmonary vein ostium (Figure 6) [36-38].

The characteristic pulmonary vein potential consists of an early far-field left atrial signal followed by an isoelectric period and then followed by the sharp near-field like pulmonary vein potential. This classic electrogram is seen when the mapping electrode is within the pulmonary vein. It follows, therefore, that if such a signal is seen on the ablation catheter, energy should not be delivered but rather the catheter pulled back until the left atrial signal is near-field and the pulmonary vein potential either not seen or appears small and far-field in nature [39].

When determination is difficult because of prior ablation and it is not clear which signal is near-field, the proceduralist can pace from the distal electrode.  If the potential following the isoelectric period (pulmonary vein potential) disappears or is 'sucked in' to the pacing spike, then one is capturing the pulmonary vein potential, and, therefore, the catheter is in the pulmonary vein and energy should not be delivered. While continuing to pace, the ablation electrode can be pulled back until the pulmonary vein potential is released and only the left atrium is captured. This signifies that the electrode has now crossed the pulmonary vein ostium and is located in the left atrium.

### Arterial Damage with Left Atrial Ablation

As part of the general ablation procedure for persistent AF or when specifically ablating atrial flutters that involve the myocardium between the left-sided pulmonary veins and the mitral annulus, linear ablation across this mitral or left-sided atrial isthmus may be required. The coronary veins and the left circumflex artery traverse in the region where radiofrequency energy is applied for such an ablation. In general, the artery lies more ventricular to the vein within 2-3 cm of the coronary sinus ostium, but beyond this, the vein may either lie adjacent to or ventricular to the adjacent artery.  Thus, ablation energy should be delivered only where atrial electrograms are seen and in a relatively atrial or posterior orientation in the right anterior oblique projection. Further compounding the possibility of arterial damage is the not infrequent necessity of ablating within the coronary sinus to offer an epicardial vantage point to create a transmural lesion across this left-sided isthmus. If the proceduralist is familiar with ultrasound imaging and the artery is visualized, this can be used as a guide. However, coronary angiography is preferable to be relatively more certain that ablation is not being done very close (<5 mm) to a significant artery.

**Figure 7:**
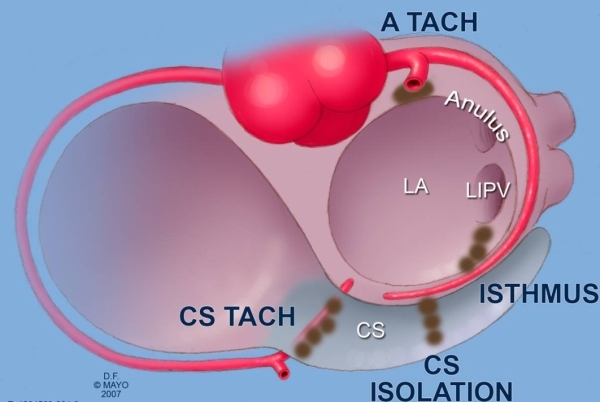
Arterial damage may occur at several locations when ablating in the left atrium for atrial fibrillation.  The circumflex artery may be damaged during left atrial isthmus ablation or attempts at coronary sinus isolation. Anterior mitral annular tachycardia ablation or coronary cusp atrial tachycardia ablation may injure the proximal left coronary arterial system.  Finally, when ablating in the proximal coronary sinus, distal branches of the right coronary artery are at risk.

Other anatomic sites where arterial damage may occur includes the proximal coronary sinus (middle cardiac vein or posterior lateral branches of the right coronary artery), epicardial Bachmann's bundle ablation (posterior wall of aorta), ablation deep in the left lower pulmonary vein (descending aorta), or in the aortic cusps for a focal atrial tachycardia (left main coronary artery or proximal left anterior descending artery) (Figure 7).

### The Esophagus

**Figure 8:**
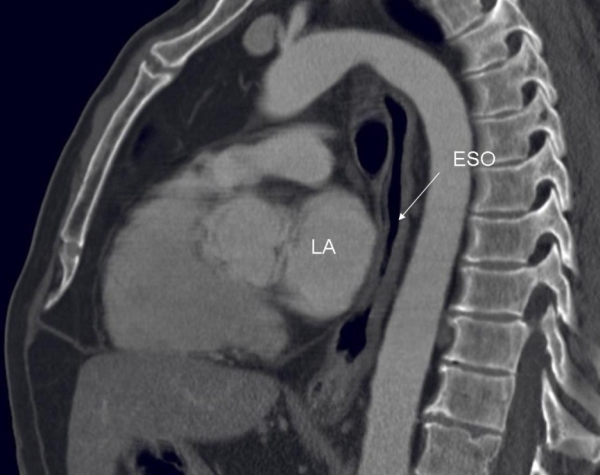
Computerized-tomographic image showing a sagittal view supposing the esophageal/left atrium relationship.  Note in this patient, a large left atrium abuts and indents into the anterior wall of the esophagus.  LA - left atrium; ESO - esophagus

Anatomically, the esophagus lies immediately posterior to the left atrium, separated only by the oblique sinus of the pericardial space and variable amounts of fat, lymphatic tissue, and parts of the autonomic nervous system (Figure 8). As a result of this anatomic proximity, the esophagus is susceptible to thermal injury during ablation. The resulting complication is atrioesophageal fistula formation - one of the most devastating complications unique to left atrial ablation procedures. This complication must especially be watched for in patients with relatively small hearts and without hypertrophied atria as may occur in the pediatric population.

Anatomic factors that are important to appreciate to try and avoid this complication include:

- Temperature probes placed within the esophageal lumen may underestimate thermal injury to the anterior wall of the esophagus because of the significant thickness of the anterior esophageal wall [40].
- The arterial vasculature of the esophagus courses often anterior to the esophagus and injury with resulting infarction of the esophagus may occur even without endoluminal temperature rise.
- The position of the esophagus relative the right or left-sided pulmonary veins is highly variable from patient to patient and within the same patient dependent on body position and peristalsis [41].
- The oblique sinus is a pericardial reflection between the parietal and visceral layers of the pericardium that gives rise to a cul-de-sac behind the left atrium. Because energy delivery within the left atrium must traverse the oblique sinus to cause damage to the esophagus, either temperature monitoring probes or direct visualization with intracardiac ultrasound of the oblique sinus can be helpful in avoiding trauma to the esophagus. With intracardiac ultrasound done from the anterior right atrium, the left atrial wall, oblique sinus, and the esophagus can be visualized in a single view. As energy is being delivered, changes in the echo density (tissue lesion creation) can be monitored and energy delivery stopped before the lesion reaches the oblique sinus and/or the anterior wall of the esophagus [42].
  It is also important to realize that trauma to the esophagus may be latent and ulceration along with esophageal perforation often occurs before or without actual perforation of the left atrial wall. Thus, there is no early evidence of atrial perforation, but rather digestive juices exit the esophagus after perforation of this structure has occurred and then invade the oblique sinus producing a fistula to the pericardial space and subsequent digestion and perforation of the posterior left atrial wall [41].
- Even more diligence is required to avoid esophageal trauma when ablating within the oblique sinus or when targeting extracardiac structures such as the retroatrial cardiac ganglia [43].
- The electrophysiologist should be aware that anatomically, there can be a paucity of myocardium in the posterior left atrium (sinus venosus derivation) between the pulmonary veins. As a result of this absence of myocardium, any ablation energy delivered there unnecessarily is more likely to cause extracardiac damage. The simple precaution of making sure that atrial electrograms are present before ablating at any site on the posterior wall rather than blindly 'connecting the dots' with a mapping guidance system will avoid unnecessary risk of trauma with no discernible benefit.

## Outflow Tract Ventricular Tachycardia

In both the adult and especially the pediatric population, accurate knowledge of the complex terrain - outflow tracts - is essential when mapping as well as ablating tachycardia from this region.

### Anatomically Overlapping Structures

It is important for the electrophysiologist to realize that because of multiple electrically active anatomic structures that overlap in the outflow tract region, one cannot assume that successful ablation or mapped electrograms performed in one region necessarily implies the origin of the electrogram or the arrhythmogenic substrate was from the mapped siten [44,45].

**Figure 9:**
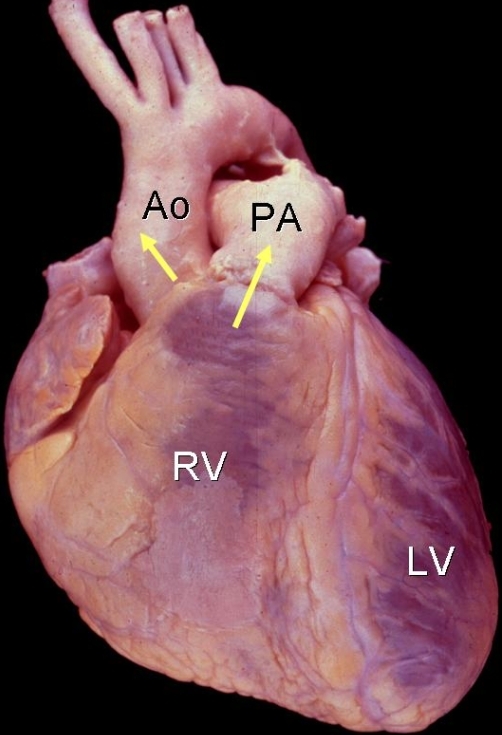
Anterior view of the heart showing the important overlapping nature of the outflow tract course.  Note the right ventricular outflow tract and pulmonary artery lie anterior and to the left of the left ventricular outflow tract and aorta.  LV - left ventricle; RV - right ventricle; PA - pulmonary artery; Ao - aortic valve.  (Figure courtesy of Dr. William D. Edwards)

The right ventricular outflow tract (RVOT) crosses the left ventricular outflow tract (LVOT) anteriorly such that the pulmonary valve lies to the left and anteriorly to the aortic valve [33,46] (Figure 9).

The right coronary cusp (RCC) of the aortic valve is directly posterior to the thick posterior infundibular portion of the RVOT.  The true septum of the RVOT is not leftward but rather posterior and similarly, the septal portion of the LVOT is its anterior portion, just behind the RVOT. Thus, a catheter placed in the RCC will record a large amplitude ventricular electrogram, the origin of which is mainly the right ventricular myocardium and partly the supravalvar left ventricular myocardium [8,47].

Recordings from the left coronary cusp (LCC) may map a supravalvar left ventricular myocardium, portions of the distal peripulmonary valve, posterior right myocardium, as well as the mitral annular left ventricular myocardium.

The noncoronary cusp (NCC) of the aortic valve generally is surrounded only by atrial structures, and thus, mapping in the NCC will identify predominately atrial signals that may arise either from the right atrium, left atrium, or the interatrial septum.  Therefore, ablation in the NCC is rarely required for ventricular tachycardia, but more often for atrial tachycardias from these regions.  However, supravalvar posterior left ventricular tachycardias can occasionally be ablated with a catheter placed in the depths of the NCC [46,48].

### Avoiding Arterial Injury

When ablating in the supravalvar portion of the LVOT, one is naturally and intuitively cognizant of avoiding cannulation of the coronary artery ostia and delivering energy there. However, anatomically, the left main coronary artery is closer and thus more susceptible to thermal injury when delivering energy in the posterior RVOT.  This is because of the anatomic overlap described above of the outflow tracts and the fact that the pulmonary valve is cephalad to the aortic valve.  Further, the pulmonary artery and distal RVOT lies to the left of the LVOT.  These anatomic facts along with the typical ostial takeoff and course of the left main coronary artery occurring cephalad to the LCC and anterior and to the left of the aortic root makes this structure very susceptible to injury when high energy ablation is done posteriorly close to the pulmonary valve in the RVOT.  Coronary angiography or direct visualization with intracardiac ultrasound can help decrease the possibility of this complication.

Electrogram interpretation may also be helpful since in the RVOT mid-portion anteriorly, atrial signals will not be seen. However, when mapping close to the pulmonary valve posteriorly, if a significant far-field atrial electrogram is seen, this likely represents origin from the neighboring left atrial appendage (LAA) [46,49,50].  The junction of the LAA and posterior leftward RVOT is often where the left main coronary artery bifurcates and the ostium of the left anterior descending artery resides.

### The Conduction System and Autonomic Innvervation

The infra-atrioventricular valvar components of the conduction system are susceptible to injury when ablating in the outflow tracts:

- The penetrating bundle of His is consistently located in the membranous portion of the intraventricular septum. The membranous septum is formed on the right side where the anterior and septal leaflets of the tricuspid valve merge and on the left, at the commissure between the NCC and RCC. Thus, damage to the atrioventricular conduction access may occur when ablating anteriorly and to the right in the NCC, posteriorly in the RCC, or in the very proximal RVOT where it merges with the inflow portion close to the membranous septum [46].
- Remnants of the infrahisian conduction system may be found in the outflow tracts (dead end tracts). When present, these structures may make mapping and specifically pace mapping of ventricular arrhythmia confusing) [33,51].  Similar to when mapping or pacing is done near the His bundle, high output pacing may capture the conduction system in addition to the ventricular myocardium, producing a different morphology than when pacing at low output and only myocardial or only conduction tissue capture is occurring. Therefore, different morphologies may be seen even though the pacing location has been kept constant. This may mistakenly give the notion that one is not at the site of origin for the arrhythmia being mapped when pacing at an inappropriate output.

The aortocaval ganglion, an important structure for both atrial as well as ventricular arrhythmia, is located in the crevice between the ascending aorta and the superior vena cava. Signals from this structure may be mapped, and ablation may purposely or inadvertently be done off this ganglia when ablating supravalvar arrhythmia [52,53].

## Conclusion

Three common arrhythmias serve as the template to help the student of electrophysiology appreciate how knowledge of the regional anatomy can help guide safe and effective ablation.

## Figures and Tables

**Figure 10:**
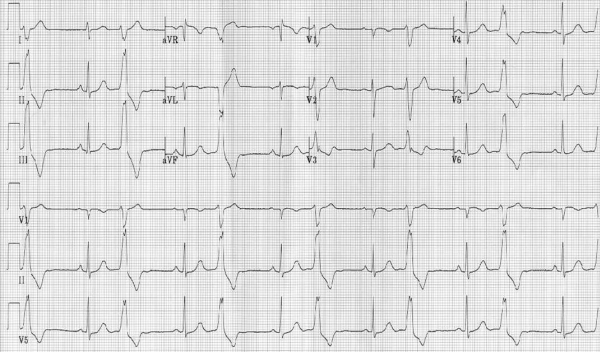
12-lead electrocardiogram from a young female with frequent ventricular ectopy and likely resulting cardiomyopathy.  At first glance, the electrocardiogram appears typical of right ventricular outflow tract tachycardia.  However, this patient had three failed prior ablations.  Note the small r wave in lead V1.  An R wave in V1 also results when ectopy origin is from the posterior right ventricular outflow tract or right coronary cusp.  Constant correlation between anatomy, electrocardiography, and fluoroscopy are essential for safe ablation in this region, particularly in children.
